# Calcium sulfate induced versus PMMA-induced membrane in a critical-sized femoral defect in a rat model

**DOI:** 10.1038/s41598-017-17430-x

**Published:** 2018-01-12

**Authors:** Yun-fei Ma, Nan Jiang, Xiang Zhang, Cheng-he Qin, Lei Wang, Yan-jun Hu, Qing-rong Lin, Bin Yu, Bo-wei Wang

**Affiliations:** 1 Department of Orthopaedics and Traumatology, Nanfang Hospital, Southern Medical University, 1838 Guangzhou Avenue North, Guangzhou, 510515 People’s Republic of China; 2 Guangdong Provincial Key Laboratory of Bone and Cartilage Regenerative Medicine, Nanfang Hospital, Southern Medical University, Guangzhou, 510515 People’s Republic of China

## Abstract

Aimed to investigate the characteristics of CS-induced membrane in comparison with the PMMA-induced membrane. Cellular components, histological changes, growth factor expressions of IL-6, VEGF, BMP-2, and TGF-β1 in the two induced membranes were compared at 2, 4, 6 and 8 weeks, respectively. We also compared the histological changes at the bone defects between CS and PMMA groups. The structural characteristics of induced membrane were similar between CS and PMMA. Endochondral ossification took place in the CS-induced membrane at 8 week. Levels of VEGF, BMP-2 and TGF-β1 in CS-induced membrane were insignificantly higher than those in PMMA-induced membrane at different time points. The expression of IL-6 was significantly higher in PMMA-induced membranes at 2nd week. In addition, osteogenic and neovascular activities of induced membranes increased with time and peaked at 6 weeks. CS promoted endochondral ossification at the broken ends of the bone defect than PMMA did. CS-induced membrane has a better capacity of generating VEGF, BMP-2 and TGF-β1.osteogenic and neovascular activities achieve highest level at 6 week. CS may have the potential to replace PMMA as a novel spacer in Masquelet technique.

## Introduction

Clinical management of critical-sized segmental long-bone defects resulting from severe trauma, surgical excision of tumour and debridement after posttraumatic chronic osteomyelitis or infected non-union poses a major problem. Segmental bone defects can be managed by autologous bone graft (ABG) as well as by adequate soft tissue envelope. Vascularized fibular graft and bone transportation with Ilizarov external fixation are two widely used techniques in the treatment of defects exceeding 5 cm^1^. Vascularized bone graft needs specialized microvascular anastomosis, and is prone to stress fracture, complications at the donor site, incomplete bone healing and risk of resorption^1,2^. Ilizarov bone transportation also has such disadvantages as pin tract infection, joint stiffness, neurological injury, irregular lines and so on^3^.

A more novel method, developed by Masquelet *et al*.^4^, utilizes the body’s natural foreign body reaction and fresh autologous cancellous bone graft to successfully repair massive diaphyseal defects in human patients. Masquelet technique involves a two-step procedure^5,6^. During the first step, a bone defect is filled with PMMA to induce formation of an encapsulation membrane. The second step starts after 8 weeks or so to reconstruct the defect. The PMMA is removed and the cavity is filled with autologous morselized cancellous bone. Of all the roles played by PMMA in Masquelet technique, like adding stability and hindering fibrous growth, acting as a foreign body to induce formation of a vascularized membrane is perhaps the most significant. This is the reason why Masquelet technique is known as the induced membrane technique. The induced membrane can prevent the graft from resorption and create a favorable micro-environment for vascularity and corticalization, promoting bone formation. Since 1986, the induced membrane technique has been widely used to manage bone defects at the tibia, femur, humerus, hand, ulna, mandibular and elsewhere^1,7–11^.

PMMA cement, introduced by Buchholz and Engelbrecht in 1970^12^ for localized antibiotic delivery, is a widely used spacer because of its benefits of being able to bear weight and variable antibiotic elution rates. It also bears such shortcomings as limited antibiotic release, incompatibility with many antimicrobial agents, and a need for surgical removal of the non-biodegradable cement before surgical reconstruction of the bone defect^13,14^. Therefore, extensive research pursuits are targeting alternative, biodegradable materials to replace PMMA, including calcium sulfate (CS)^14,15^.

CS has benefits of good biological compatibility, osteogenic property and osteoconduction^16^. Recently it has been found to be potentially osteoinductive^17^, inducing differentiation of bone marrow mesenchymal stem cells into osteoblasts. Due to its complete biodegradability, an additional significant advantage over PMMA, the use of CS as a delivery vehicle has been investigated^18^. It has also been widely used in clinic as a local antibiotic carrier for the treatment of chronic osteomyelitis, because it is totally absorbed over a period of several weeks, releasing the entire antibiotic load^19^. We found that it also induced formation of membrane-like structure in clinical cases^20^, but the histological and biochemical characteristics of the CS-induced membrane were unclarified. There has been no research addressing these problems as well as the differences between the PMMA-induced versus the CS-induced membranes in repair of large segmental bone defects.

Therefore, we hypothesized that PMMA might be replaced by CS since they can both accomplish the important role of inducing membrane and the major disadvantage of PMMA might be overcome and made up for by the major benefit of CS. The degradability of CS may allow one-stage reconstruction of large segmental bone defects, sparing the necessity of surgical removal of the spacer.

As the first attempt to testify our hypothesis, the present study was aimed to characterize CS-induced membrane and make comparisons between the CS-induced and PMMA-induced membranes in repair of large segmental femoral defects in a rat model.

## Materials and Methods

### Animal care

Male Sprague-Dawley rats (n = 60; Guangzhou, Guangdong, China), weighing approximately 260–280 g were randomized into two experimental groups and one empty control group (n = 20, each). Critical-sized femoral defect was created in all rats. In experimental groups filled with CS (Stimulan Rapid Cure, Biocomposites, Keele, U.K.) (CS group), and PMMA (Simplex P, Stryker, Kalamazoo, MI) (PMMA group), respectively. Nothing in the control group. The general anesthetic used was sodium pentobarbital. Iidocaine was used for additional regional anesthesia before osteotomy. The research was conducted in accordance with the Declaration of Helsinki and with the Guide for Care and Use of Laboratory Animals. All animal experimental procedures and the animal use and care protocols were approved by the Review Committee on Ethical for the use of Human or Animal subjects of NanFang Hospital (Project SYXK No. 2015-0056, Southern Medical University, Guangzhou, Guangdong, China).

### Surgical procedure

After intramuscular injection of sodium pentobarbital (3%, 1 ml/kg body weight), all rats were shaved and placed on a heating blanket during surgery. After the superficial fascia and muscles of the right hind limb were incised, exposing the lateral aspect of the femoral bone. A six-hole, 1.0 mm stainless steel mini-plate (BIORTHO, Jiangsu Province, China) was applied to the femur shaft and secured in place with two proximal and two distal 1.1 mm cortical screws (BIORTHO, Jiangsu Province, China). A critical-sized defect (CSD) measuring 10 mm was created in the femur bone shaft, beneath the plate, using a high-speed power drill. The bone defects were filled with CS or PMMA cylinders (2.8 mm in diameter, 10 mm in length) respectively^21^. The wounds were closed with interrupted 4-0 Vicryl sutures. Analgesia was given for 3 days postoperatively. Penicillin 20,000 IU/kg was administered intramuscularly immediately, 24 and 48 hours after operation.

Five animals in each group were sacrificed each time at 2, 4, 6 and 8 weeks to harvest the membranes surrounding the CS/PMMA spacers for analysis. Subsequently the animals were euthanized with an overdose anesthetics administered intraperitoneally.

The membranes were divided into four fractions which were respectively fixed in 10% formalin solution for later paraffin embedding, shock frozen (−80 °C) for later PCR analysis, shock frozen for later Western-Blotting analysis, or fixed in 10% formalin solution for further immunohistochemistry (IHC) analysis.

### Digital radiographs

Digital radiographs were taken immediately, 2, 4, 6 and 8 weeks post-operation under anesthesia using an Oralix AC Densomat X-ray machine (Gendex Dental System, Milan, Italy). The femur samples were harvested at 2, 4, 6 and 8 weeks postoperatively for histological examination and micro-CT scanning.

### Micro-computed tomography (Micro-CT)

The morphology of the newly formed and reconstructed femur bone were using an animal micro-CT scanner (Skyscan1176, Bruker Micro-CT, Belgium). The Scanning parameters with an 80 kVp energy setting, intensity of 313 uA with 280 ms acquisition time and a voxel resolution of 18 µm. The defective region was identified by a contour as a traced region of interest (ROI), the relative measurements of bone mineral densities (BMDs) and bone volume/total volume (BV/TV) were calculated.

### Histological observation of bone defects

After micro-CT scanning, the femur samples were decalcifed in 10% Ethylene Diamine Tetraacetic Acid (EDTA, pH = 7.4) and dehydrated in graded ethanols (70–100%). Finally, they were embedded in paraffin and cut in 5 µm thick sections for staining. Masson’s Trichrome and Safranin O-fast green staining were utilized according to the manufacturer’s protocols.

### General histological observation of induced membrane

The harvested samples were embedded in paraffin and cut in 5μm thick sections for histological staining. Stained with Hematoxylin & Eosin and used to an Optiphot-2 (Olymbus@) photomicroscope to distinguish cells. The Safranin O staining, Collagen II and Aggrecan immunohistochemistry staining were carried out for further determination.

### Real-time polymerase chain reaction (Real-time PCR)

The membranes were stored in liquid nitrogen before RNA analysis. RNA was isolated according to the manufacturer’s instructions. The amount of RNA was measured as OD values on UV-1750 UV spectrophotometer by Shimadzu (Japan). Amplifications were performed with the ABI 7300 Real-Time PCR System (ABI, Carlsbad, CA, USA) with different primers. The primers used were as follows: Interleukin-6 (IL-6) forward AGACTTCCAGCCAGTTGCCTT and reverse TTGTGGGTGGTATCCTCTGTGA; Vascular endothelial growth factor (VEGF) forward TCCTGTGTGCCCCTAATGC and reverse ACGCACTCCAGGGCTTCAT; Transforming growth factor-β1 (TGF-β1) forward GACGGAATACAGGGCTTTCG and reverse CCTCGACGTTTGGGACTGAT; Bone morphogenetic protein-2 (BMP-2) forward AGAGCTTTGATGTCACCCCG and reverse GGAGACACCTGGCTTCTCCTC. The expression of each compound was related to compare the housekeeping gene GAPDH with one selected control sample.

### The content measurements of TGF-β1 and BMP-2

The membrane tissues were homogenized in lysis buffer at 4 °C, followed by centrifugation at 20000 × g for 30 min at 4 °C. TGF-β1 and BMP-2 were detected using monoclonal goat anti-rabbit TGF-β1 antibody (BA0290, Boster) and monoclonal goat anti-mouse BMP-2 (Ab6285, Abcam), respectively. Determination of GAPDH with mouse anti-GAPDH antibody (Abcam) served as a loading control. The blots were blocked (10% non-fat dried milk in 1 m M Tris, 150 m M NaCl, pH 7.4) for 1 h, incubated for 1 h at RT with primary antibody in blocking buffer with 0.5% Tween20 and 0.5% bovine serum albumin (BSA) and then incubated for 1 h with horseradish peroxidase-conjugated secondary antibody (Santa Cruz Biotechnology) diluted 1:1000 in blocking buffer with 0.5% Tween20 and 0.5% BSA at RT. The density of individual bands was determined using Fusion V software and normalized to the density of the corresponding GAPDH band.

### Statistical analysis

All data collected were reported as means with standard deviations and P values ≤ 0.05 were defined as statistical difference. For normally distributed data, Student t test or one-way analysis of variance (ANOVA) was used to compare differences between 2 different groups or among more than 2 groups. All statistical analyses were performed using SPSS 13.0 software (SPSS Inc, Chicago, IL, USA).

## Results

### The tracked X-ray radiographs of the typical models

**Figure 1:**
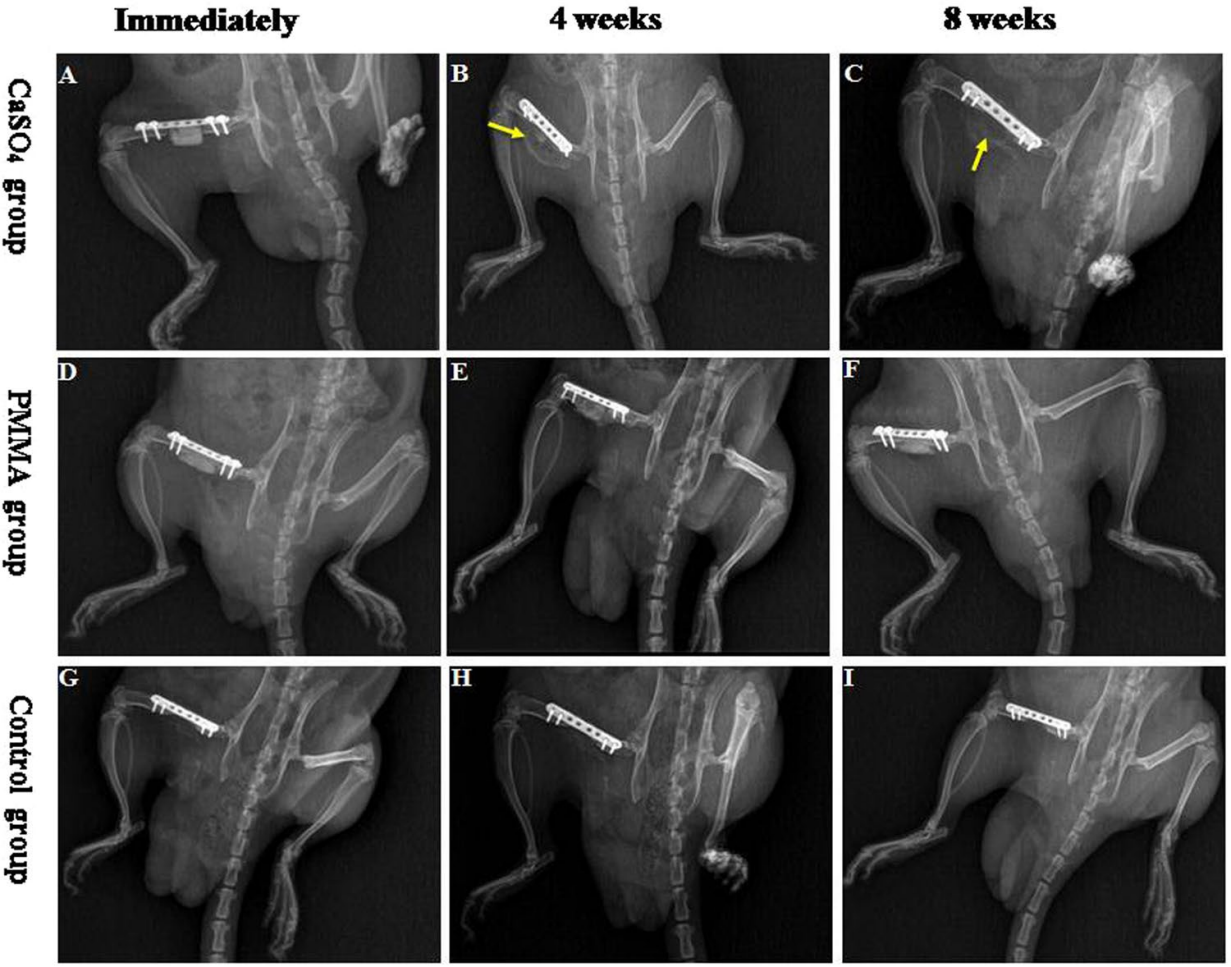
Listed X-ray images of typical rats with femur bone defect after implantation CS, PMMA and control group, respectively. The time point was immediately, 4 and 8 week postoperatively. As time changed, CS can be completely degraded and absorbed, and we can find the membranous structure (arrows indicate) around the bone defect at 4^th^ and 8^th^ week. However, PMMA and control groups have not changed.

At the immediately after surgery, as presented in Fig. 1A,D, both CS and PMMA were radiopaque so that the bone substitute and the broken ends were clearly visible.

At 4^th^ week, much of the CS appeared fuzzy, dotted and flocculent, indicating partial degradation and absorption. A membrane-like structure appeared around the bone defect while the PMMA material remained relatively clearly visible (Fig. 1B,E).

At 8^th^ week, CS was completely degraded and absorbed, membrane-like structure also appeared. The PMMA material showed no significant change compared with previous (Fig. 1C,F).

Nothing grew into the critical-sized bone defects in the empty control group at different time points (Fig. 1G–I).

### Micro-CT measurement

**Figure 2:**
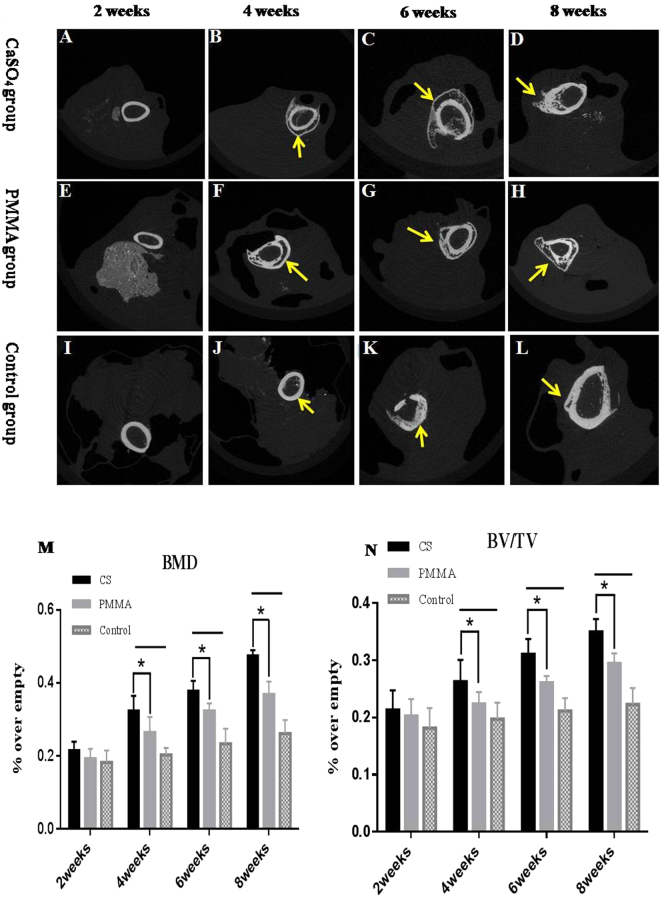
Listed cross sections analysis at 2, 4, 6 and 8 weeks. Panels(A–D) were the CS group, panels (E–H) were PMMA group, and panels(I–L) were control group. The micro-CT cross sections of the defective area showed more obvious new bone formation (arrows indicate) in CS group than in PMMA and control groups. Morphometric evaluation of (M) the local bone mineral densities (BMDs) and (N) bone volume/total volume (BV/TV). *Indicated that the PMMA group exhibited the lowest levels of both BMDs and BV/TV of the defects when compared to CS groups (P < 0.05). ^—^Indicated that the control group exhibited the lowest levels of both BMDs and BV/TV of the defects when compared to 2 experiment groups (P < 0.05). The data further revealed that CS groups resulted in significantly more BMDs and BV/TV of the defect compared to the PMMA group at 4, 6, 8 weeks (P < 0.05).

The micro-CT cross sections showed more obvious new bone formation in CS group than in PMMA group. Further compared with the empty control group, CS and PMMA groups exhibited significantly more new bone formation but there were gaps still existing in the bone defect (Fig. 2A–L).

The quantity of the newly formed bone was calculated by morphometric analysis (Fig. 2M and N). The data demonstrated significantly greater BMDs in CS group than in PMMA group at 4, 6 and 8 weeks postoperatively (P < 0.05). Compared with the control group, there were significant differences in 2 experimental groups at 4, 6 and 8 weeks postoperatively (P < 0.05). However, there was no significant difference among the 3 groups at the 2^nd^ week postoperatively (P = 0.135). The BV/TV results exhibited a similar trend. The mean BV/TV percentage in CS group was significantly higher than that in PMMA group at 4, 6 and 8 weeks postoperatively (P < 0.05), but there was no significant difference at the 2^nd^ week among the 3 groups (P = 0.59).

### Histological analysis of bone defects

**Figure 3:**
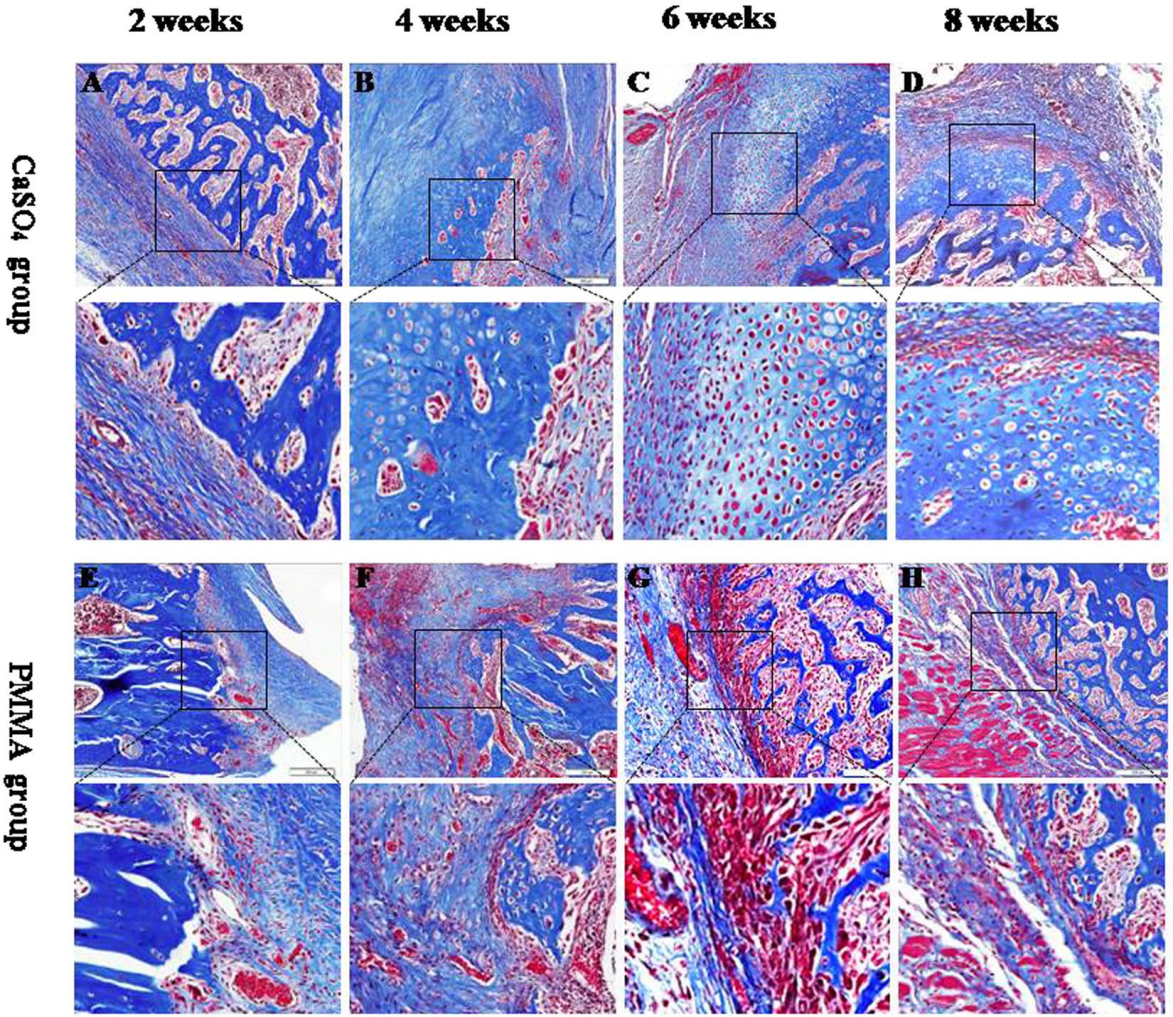
(**A–D**) and (**E–H**) representative histological sections of the end of bone defect with Masson’s Trichrome staining the histological changes over the course of maturation of the endochondral ossification (black-box indicate) in CS group and PMMA group after 2, 4, 6, 8 weeks of implantation, respectively (original magnification  ∗ 200).

Through Masson’s Trichrome staining, we can find the following phenomenons (Fig. 3).

At the 2^nd^ week, we found lots of blue-staining fibrous tissues, lymphocytes, macrophages and micro-vascular sections in both CS and PMMA groups. The broken ends presented even and no new bone tissue formed (Fig. 3A and E).

At the 4^th^ week, there were more blue-staining fibrous tissues and micro-vascular sections in both CS and PMMA groups. The lymphocytes and macrophages reduced significantly in number compared with those at the 2^nd^ week. The diameter of vascular section increased obviously compared with previous. A small amount of new bone formed at the broken ends (Fig. 3B and F).

At the 6^th^ and 8^th^ weeks, more fibrous tissues and vascular sections were seen. A few lymphocytes and macrophages were also apparent. Obvious endochondral ossification was seen at the broken ends. Much more new bone tissue formed near the ends (Fig. 3C,D and G,H).

**Figure 4:**
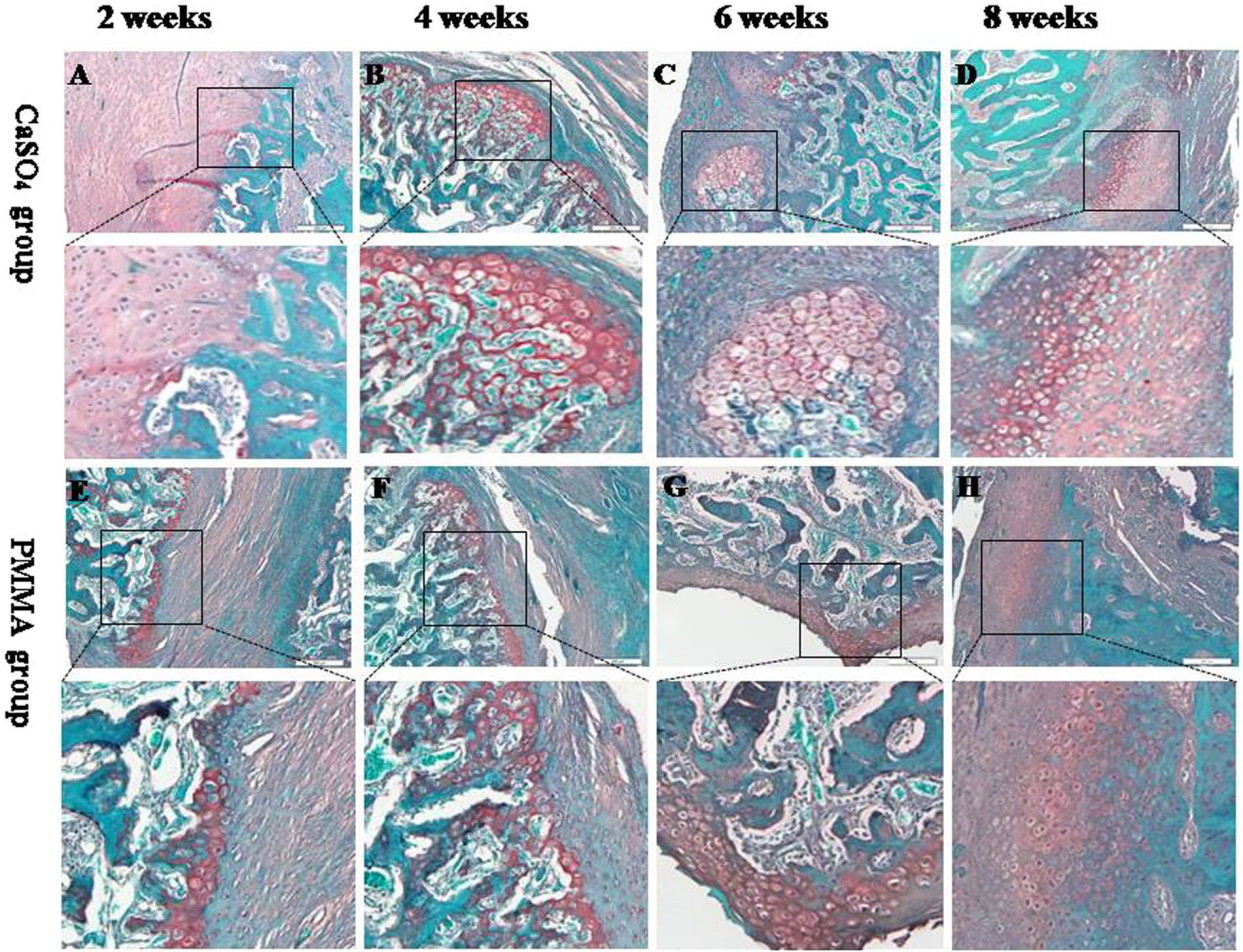
(**A–D**) and (**E–H**) representative histological sections of the end of bone defect stained with Safranin O and fast green demonstrating the histological changes over the course of maturation of the endochondral ossification (black-box indicate) in CS group and PMMA group after 2, 4, 6, 8 weeks of implantation, respectively (original magnification  ∗ 200).

At the same time, the histological sections of the end of bone defect stained with Safranin O and fast green demonstrating the similar results to those of Masson’s Trichrome staining (Fig. 4).

### Gross observation and radiographic analysis of the induced membrane

**Figure 5:**
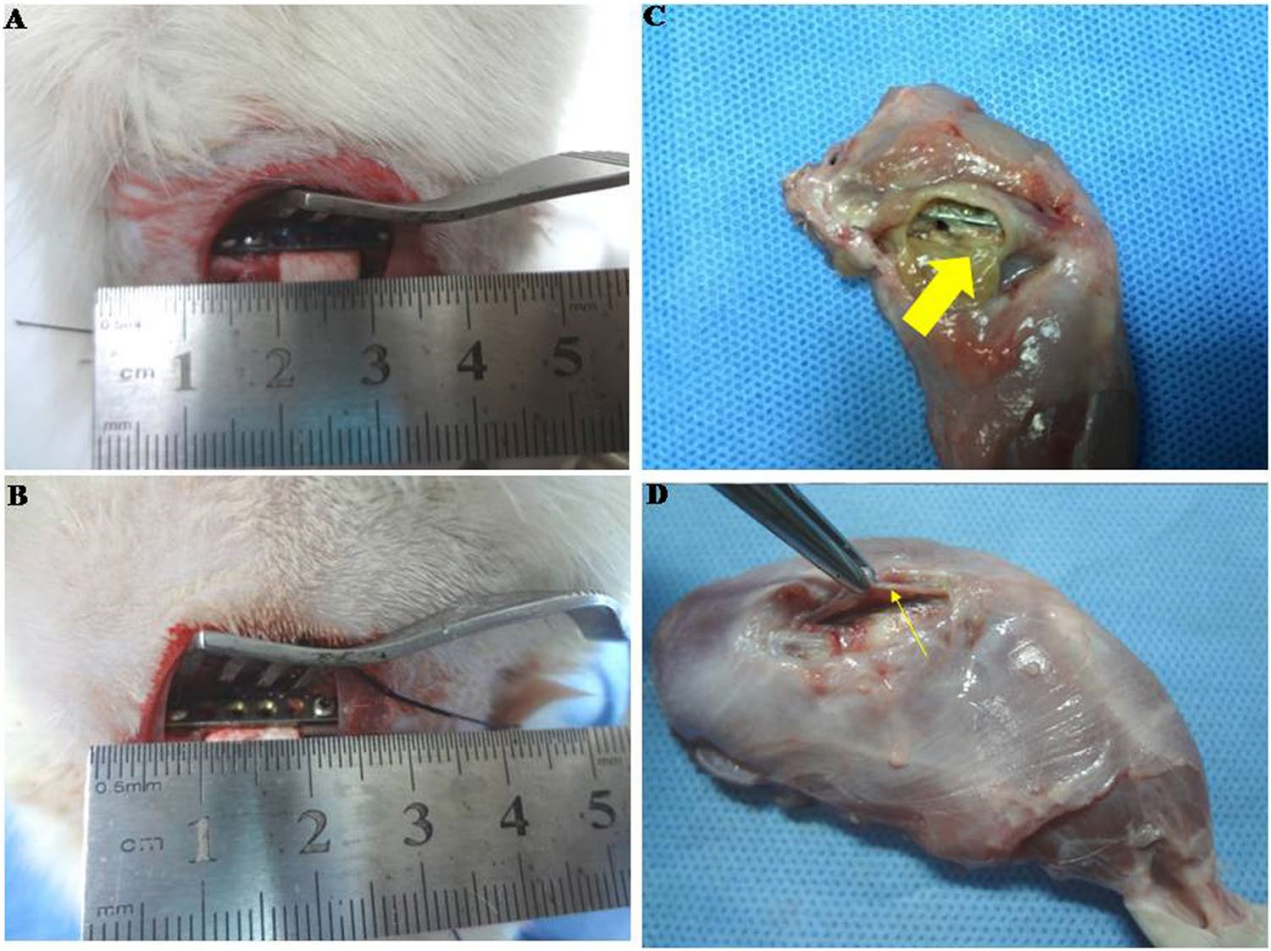
Induction, harvesting and macroscopic appearance of membranes induced at bone defects: the stabilized femur bone defect, which is filled with CS/PMMA cement (**A,B**) and the wound is closed; after 2, 4, 6 and 8weeks, the animals were killed and the femurs with induced membranes were harvested; Thick yellow arrow (**C**) indicates the induced membrane by CS; Thin yellow arrow (**D**) indicates the induced membrane by PMMA.

Gross observation showed the following macroscopic appearances of the induced membranes at the bone defects filled with CS/PMMA cement for 2, 4, 6 and 8 weeks (Fig. 5).

CS group. At 2^nd^ weeks, a thin soft membrane-like structure formed and adhesive to the surrounding muscle tissues. The outer layer of membrane was dark red fibrous tissue-like structure, and the inner layer was smooth and light green. Slight degradation of the CS was observed in the bone defect. The smooth light green inner layer did not adhere to the CS. At 4^th^ weeks, the induced membrane was gradually thickening. The outer layer of membrane was still dark red, but a clear border appeared between the membrane and surrounding muscle tissues. The CS partly degraded in the bone defect. Some clear yellow-green liquid was found in the bone defect. The inner layer of the membrane became yellow-green. A small amount of bone callus formed around the broken ends of the bone defect. At 8^th^ weeks, the membrane thickened more and became elastic (Fig. 5C). Most of the CS degraded in the bone defect. There was an amount of clear yellow-green liquid in the bone defect. A lot of bone callus generated around the plate and the both broken ends, but the bone callus was not yet fully mature, with an obvious boundary with surrounding bone tissue.

PMMA group. At 2^nd^ weeks, around the PMMA a thin and dark red induced membrane structure formed which was similar to the periosteum, but thicker and tougher than the periosteum. The induced membrane adhered to the PMMA and its surrounding muscle tissue. No degradation of PMMA or any liquid was found in the induced membrane. At 4^th^ weeks, the induced membrane was generally thickening, and tightly adhesive to the PMMA. Some bone callus formed around the broken ends. The PMMA around the bone defect showed no significant change compared with before. At 8^th^ weeks, the induced membrane around the bone defect partly adhered to the surrounding tissues (Fig. 5D). Its thickness increased significantly. The PMMA still showed no obvious change compared with before, but some bone callus generated and surrounded the plate and both ends of the bone defect.

Control group. At 2^nd^ weeks, no membranous-like tissue was generated. The bone marrow cavity was closed, and osteosclerosis showed at both broken ends. A small amount of fibrous tissues grew gradually into the bone defect. At 4^th^ weeks, significant osteosclerosis showed at the broken ends. Lots of fibrous tissues grew into bone defect. At 8^th^ weeks, the bone defects were already filled with white fibrous tissue.

**Figure 6:**
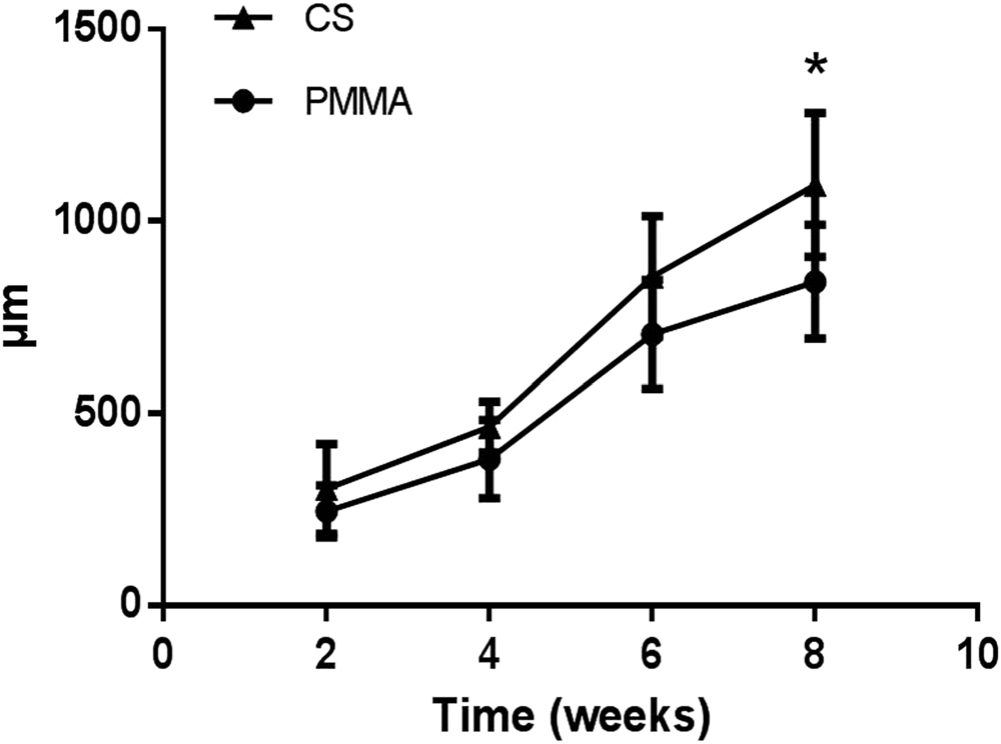
At 2, 4, 6 and 8 weeks postoperatively, as the CS-/PMMA-induced membranes matured, their thickness and fibrosity increased gradually. The thickness of CS-induced membrane was significantly greater than that of PMMA-induced membrane at 8^th^ week (p < 0.05).

As the CS-/PMMA-induced membranes matured, their thickness and fibrosity increased significantly. The CS-induced membranes were generally thicker than the PMMA-induced ones. Specifically, the thickness of CS-induced membrane (1095 μm) was significantly greater than that of PMMA-induced membrane (842 μm) at 8^th^ week (p < 0.05) (Fig. 6).

### Morphological structure of different induced membranes

**Figure 7:**
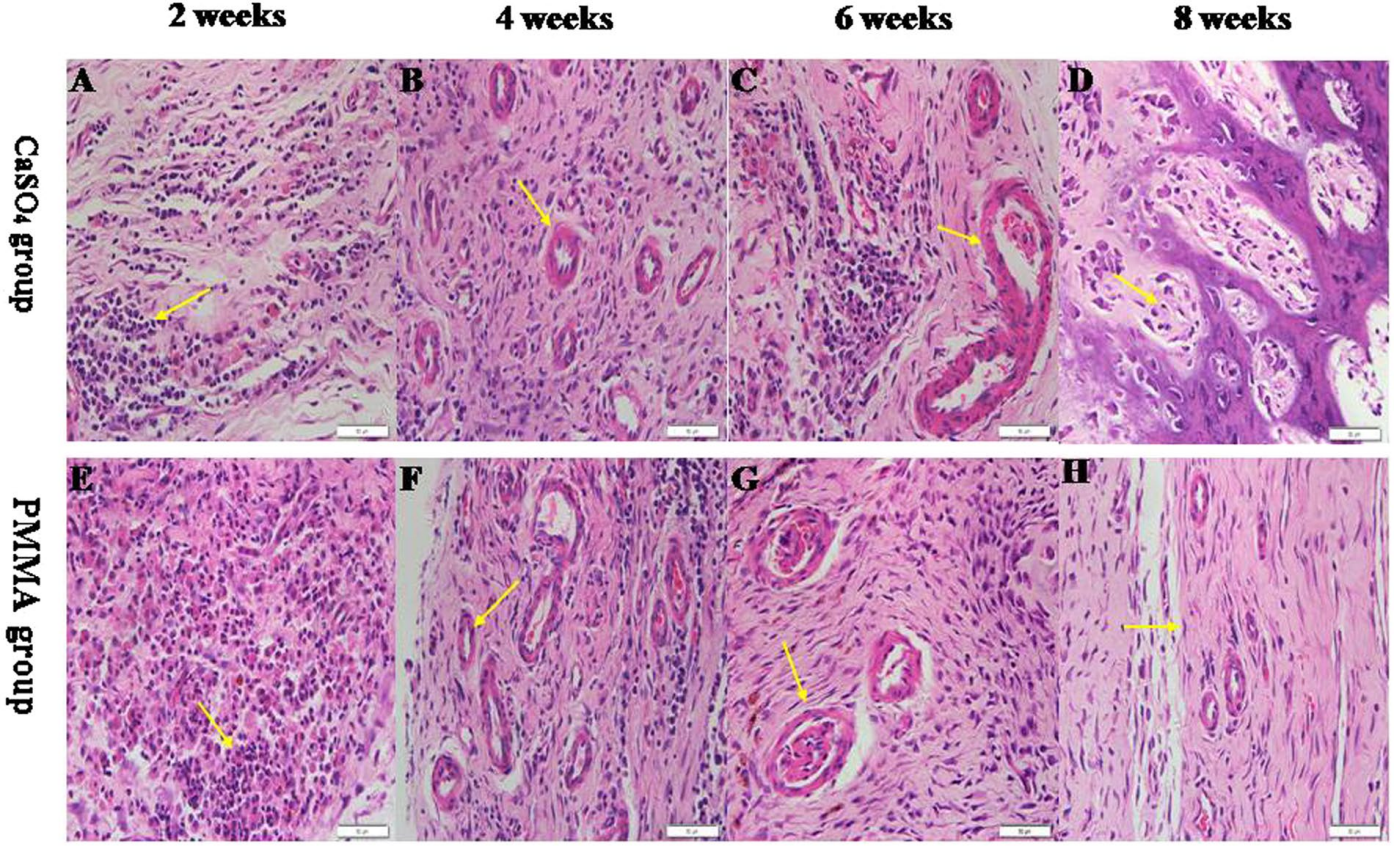
(**A**–**D**) Representative histological sections of induced membranes stained with H&E staining demonstrating the histological changes over the course of maturation of membranes induced around CS after 2, 4, 6, 8 weeks of maturation (original magnification ∗ 200). Figure 7A Some Inflammatory cells (arrow) can be seen from 2^nd^ membrane. Figure 7B Newly formed small blood vessel tissues (arrows) from 4^th^ membrane. Figure 7C Mature fibrous tissue of a 6^th^ membrane. Some larger blood vessels (arrows) are seen. Figure 7D Endochondral ossification takes place in induced membranes (arrows indicate cartilage) from 8^th^ membrane. (**E**–**H**) Representative histological sections of induced membranes around PMMA. Histological changes were similar to the CS group at 2, 4, 6 weeks of maturation, but we can not find endochondral ossification at 8^th^induced membrane.

CS group. As shown in Fig. 7A, at the 2^nd^ weeks, local and mild acute inflammatory reaction and diffuse edema were noticed, capillaries as well as neutrophils, monocytes, fibroblasts, myofibroblasts, and collagen were seen on the outer surface of the membrane, and synovial-like epitheliums were observed in the inner part of the membrane. At the 4^th^ week, the membrane was similar to that described above, and myofibroblasts and collagen fibers mostly arranged parallel to the spacer surface, but less edematous reaction appeared (Fig. 7B). At the 6^th^ week, the thickness and fibrosity increased significantly; only a handful of monocytes, neutrophils and small capillaries were observed; larger vessels developed in the outer part of the membrane (Fig. 7C). At the 8^th^ week, the edema was almost completely absorbed and multinucleated giant cells decreased in number; endochondral ossification, newly formed bone tissue and even mature lamellar bone appeared in the induced membranes (Fig. 7D).

PMMA group. At different time points postoperatively, the morphological characteristics of induced membranes around PMMA were generally similar to those around CS. The extent of inflammatory and edema reaction was more severe than in CS group at 2^th^ week. But endochondral ossification or newly formed bone tissue was not observed at the 8^th^ week (Fig. 7E–H).

### Endochondral ossification of the induced membranes in CS group at 8 weeks postoperatively

**Figure 8:**
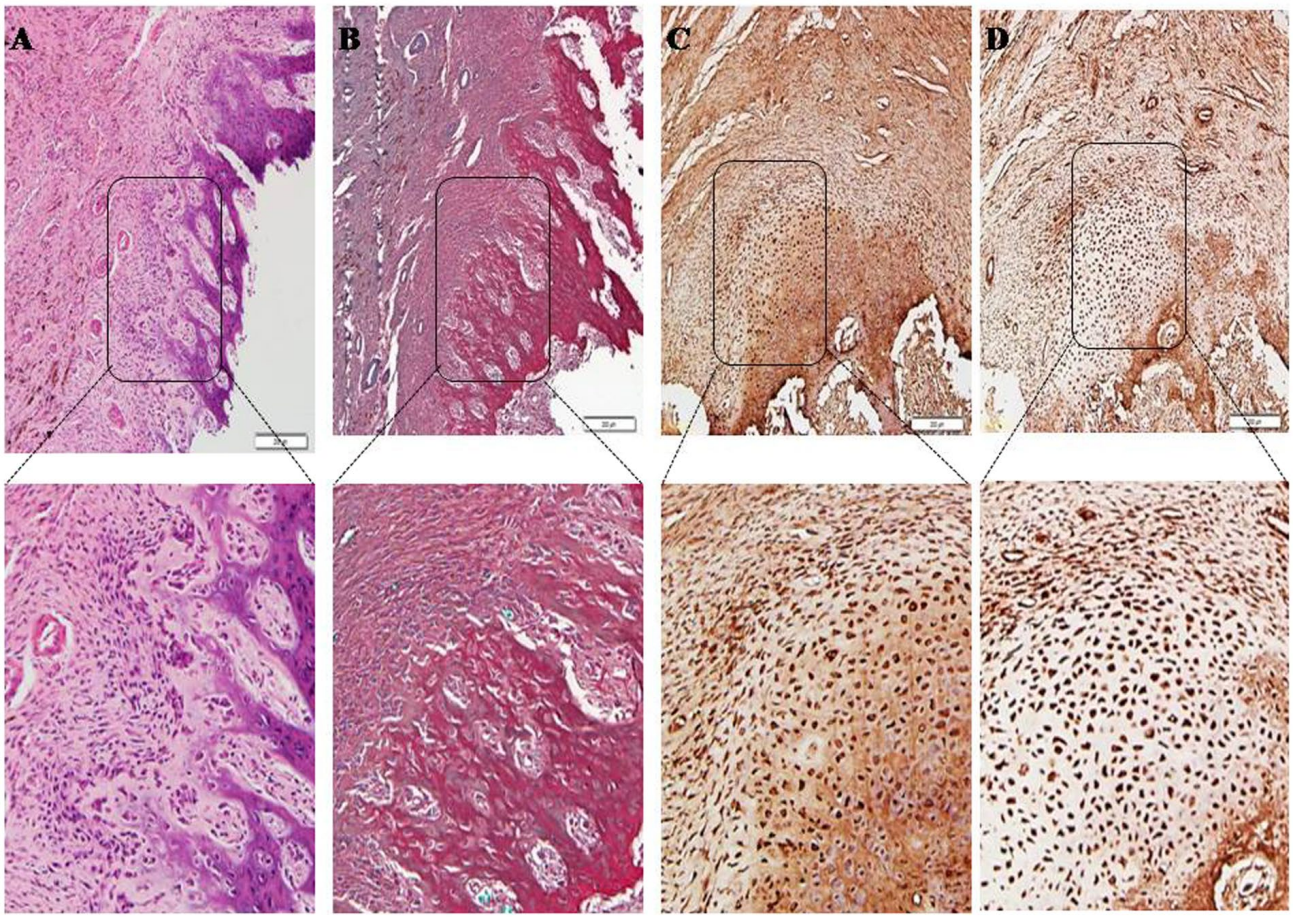
(**A**–**D**) Representative histological sections of induced membranes stained with H&E (**A**), safranin-O (**B**), Collagen-II (**C**) and Aggrecan(**D**) staining demonstrating the endochondral ossification in induced membranes (black-box indicate) from 8^th^ membrane. (original magnification  ∗ 100).

The 8^th^ week-induced membranes in CS group were further identified. H&E and Safranin-O staining confirmed endochondral ossification and newly formed bone tissue (Fig. 8A,B). IHC staining also showed multiple sites of fixation for antibodies against Collagen-II and Aggrecan, demonstrating the endochondral ossification presented in Fig. 8C and D.

### The contents of TGF-β1 and BMP-2 in the induced membranes

**Figure 9:**
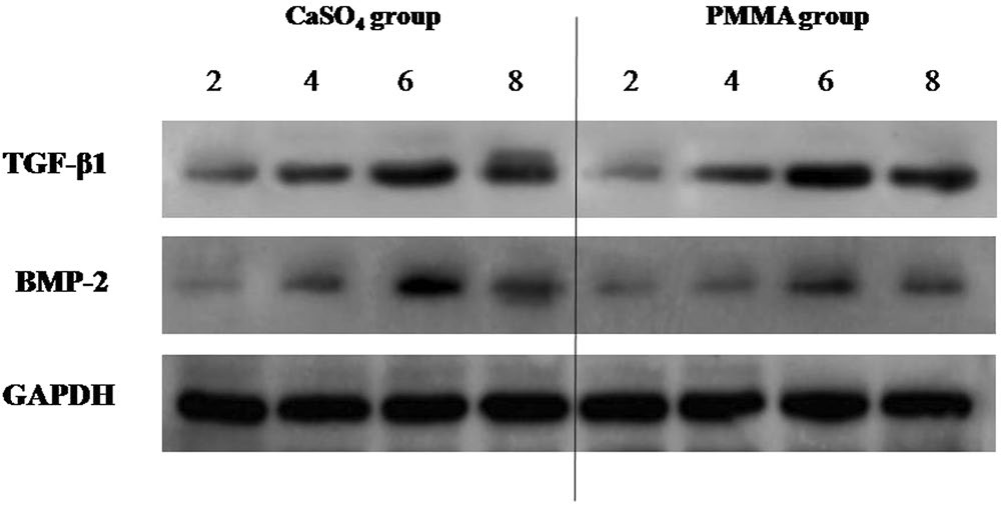
Western-blot analysis revealed the presence of TGF-β1 and BMP-2 in the induced membranes harvested from CS and PMMA groups at 2, 4 and 6 weeks. The TGF-β1 and BMP-2 protein contents went up gradually at 2, 4 and 6 weeks but declined after 6 weeks.

**Figure 10:**
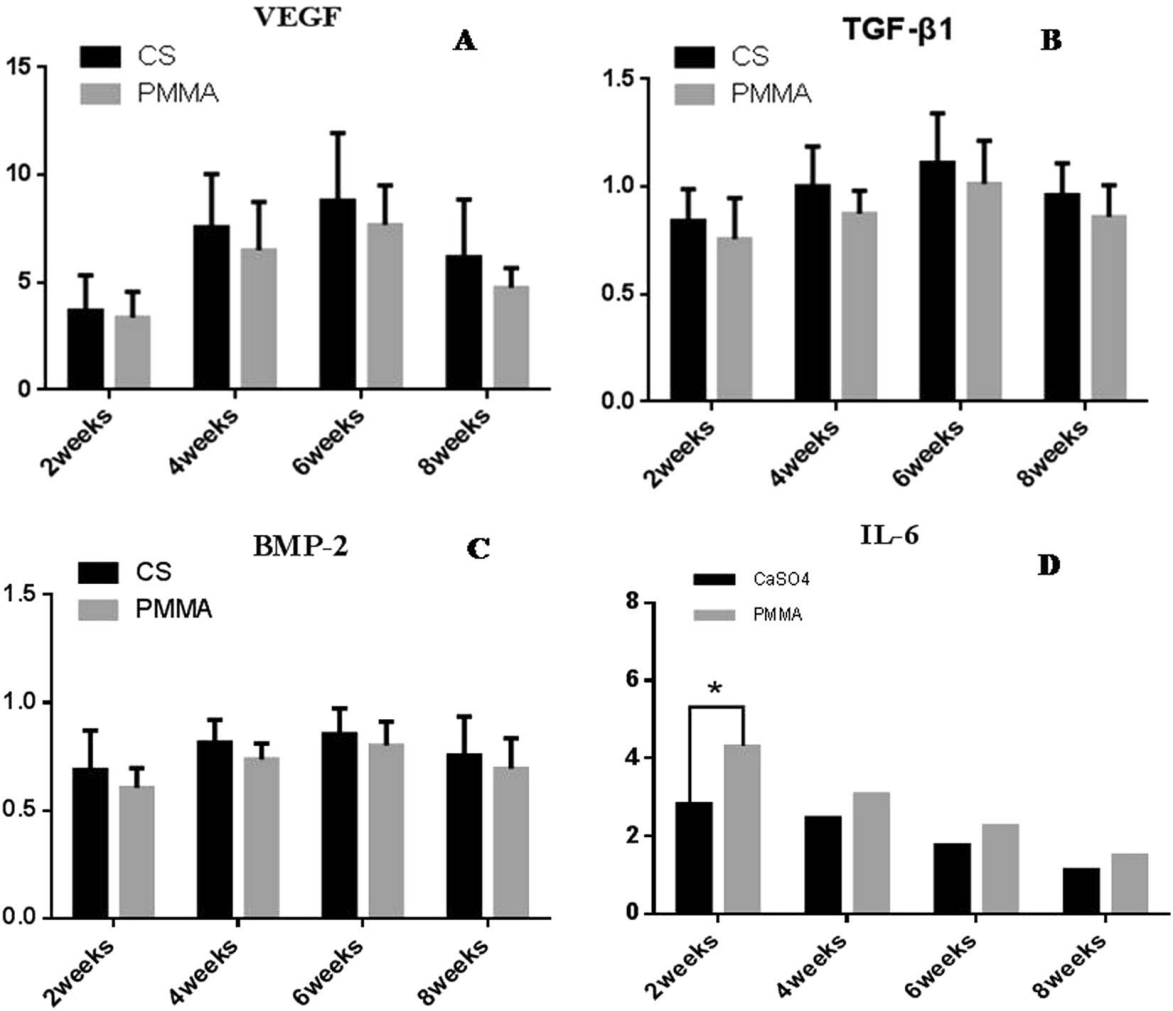
The expressions of VEGF, TGF-β1 and BMP-2 were insignificantly higher in CS-induced membranes than in PMMA-induced ones at 2, 4, 6 and 8 weeks (**A**,**B**,**C**). The expression of IL-6 showed a gradually decreasing tendency in the two experimental groups; it was significantly higher in PMMA-induced membranes than in CS-induced ones at the 2nd week but insignificantly higher at 4, 6 and 8 weeks (**D**).

The TGF-β1 and BMP-2 protein contents went up gradually at 2, 4 and 6 weeks but declined after 6 weeks (Fig. 9). The expressions of VEGF, TGF-β1 and BMP-2 were insignificantly higher in CS groups than in PMMA ones (Fig. 10A–C). The expression of IL-6 showed a gradually decreasing tendency in both groups; it was significantly higher in PMMA group than in CS group at 2^nd^ week but insignificantly higher at 4, 6 and 8 weeks (Fig. 10D).

## Discussion

Our present work has made the following four main findings. Firstly, CS as a spacer can also induce formation of a membrane which may be thicker than that induced by PMMA. Secondly, endochondral ossification may take place in the CS-induced membranes but not in the PMMA-induced membranes, suggesting that CS-induced membranes may have a real osteogenic potential to repair bone defect. Thirdly, the expressions of factors (VEGF, TGF-β1 and BMP-2) may be insignificantly higher in CS-induced membranes than in PMMA-induced ones at different time points. The contents of the factors may increase with time and peak at 4 to 6 weeks and decline gradually afterwards. Fourthly, CS may promote endochondral ossification at the broken ends at different time points better than PMMA.

Through histological staining we found that a membrane-like fibrotic capsule formed around the bone defect in CS. The inner layer of the CS-induced membrane there were synovial-like epithelial cells and a large number of vessels; fibroblasts formed on the outer layer which was full of capillaries and collagen fibers. These organizational structures were similar to those of PMMA-induced membranes. So we conclude that CS can also induce formation of a membrane structure which is similar to that of PMMA-induced membranes.

We observed the same structures in induced membranes harvested at different time points in both CS and PMMA groups. The histological changes of the induced membranes we observed were similar to the previously reported^22,23^. In addition, we believe that the greater thickness of the CS-induced membrane is a crucial factor which plays a mechanical role in preventing soft tissue protrusion and subsequently trying to slow down bone resorption. At this point, the CS-induced membrane may be superior to the PMMA-induced membrane.

We also observed some endochondral ossification in CS-induced membrane at the 8^th^ weeks. As far as we know, no one has reported this phenomenon before. We continued with safranin O staining and IHC staining of Collagen II and Aggrecan. Results further indicated the presence of endochondral ossification. CS has good biological compatibility, osteogenic property and osteoconduction^16,17^. However, some researchers have proposed that CS has potential osteoinductivity^24^. We hypothesize that a local environment of slight acid and high calcium during the degradation of CS may be the main factor promoting new bone formation and endochondral calcification. In a relatively closed environment of bone defect, an acid environment can lead to bone decalcification reaction, and cause the release of decalcified bone matrix and bone morphogenetic protein, resulting in the bone formation reaction of bone defect^25^. CS can be completely degraded and absorbed in the bone defect area, providing a source of calcium for new bone tissue, and promoting the proliferation and differentiation of osteoblasts to form new bone tissue, resulting in bone formation^26^.

Most researchers believe that the osteoinductivity of CS can promote osteogenesis^14,16,17^. CS can also fill the defect area, prevent soft tissue from growing into the bone defect, provide bone matrixes and cause endochondral ossification. With the CS absorption, new bone can gradually fill the bone defect^27^.

We observed the relative expression levels of VEGF, TGF-β1 and BMP-2 in CS-induced membranes were higher than those in PMMA-induced ones, but no significant differences were found between two groups. We also found the contents of different factors reached the maximum at the 6^th^ week, and then gradually reduced. This finding is different from that by previous studies^23,28^.

VEGF could induce angiogenesis, which played an important role in bone repair and bone regeneration. Moreover, blood vessels can provide nutrients and oxygen and play an important role in regulation of bone remodeling by attracting endothelial cells and osteoclasts and by stimulating osteoblasts differentiation.

BMP-2 can promote chemical trends, gather, differentiation of BMSCs, which can directly mediate osteogenic differentiation of BMSCs and immature osteoblasts. In our present work, the increased BMP-2 production in the membranes might have played a role in the osteogenic differentiation of BMSCs.

TGF-β1 can also promote proliferation and differentiation of BMSCs, help synthesis of extracellular matrix protein and regulate the immune function^29^. TGF-β1 has been shown to play a central role in foreign body encapsulation. Therefore, TGF-β1 has been considered as a putative regulator of osteoclastic-osteoblastic interaction and is continuously synthesized in induced membranes^21,30^.

The expression of IL-6 showed a gradually decreasing tendency. It was significantly higher in PMMA group than in CS ones at 2^nd^ week. This might have been related to the PMMA exothermic inflammation reactions.

The induced membrane plays an important role in bone repair and healing. Despite the significant differences we measured between CS and PMMA membranes induced around the bone defects, it is interesting to note that both of the induced membranes can promote bone healing.

We observed a significant increase in the BMDs and BV/TV of the bone defect from 2 to 8 weeks in the two groups. We also found more obvious new bone formation in CS group than in PMMA group through the structural characteristics. Compared with previous studies on PMMA-induced membrane, CS-induced membranes also contained well-vascularized fibrous tissue that was mostly preserved during follow-up. And most importantly, we found some intramembranous ossification in the CS group specimens. We speculate that CS might provide a source of calcium for new bone tissue, and promote proliferation and differentiation of osteoblasts to form new bone tissues.

Being aware that our observations in a rat model cannot be extrapolated directly to human conditions, we believe that CS may replace PMMA in that it promotes bone growth but does not require a secondary surgery in Masquelet technique. Therefore, the healing process would capitalize on all this angiogenic and osteogenic activity and thus could result in a much quicker healing than the PMMA does.

Our present study also has a major limitation. We compared the histological and biochemical characteristics of the membranes induced by CS and PMMA. It is only a preliminary proof that CS can induce membranes, but further experiments of molecular biology are required to verify and compare the differences between CS- and PMMA-induced membranes. Further animal experiments are necessary to verify our hypothesis that CS might be used in one-stage reconstruction of segmental bone defects, sparing surgical removal of the spacer.

## Conclusion

The membranes induced by CS are similar to those of PMMA in many respects. The osteoinductivity of CS is significantly better than that of PMMA. Thus, to maximize the bone-healing effect, we propose that PMMA can be replaced by CS early (6 weeks) when the osteogenic activity of CS is at the peak. Since CS has benefits of good biological compatibility, osteogenic property, osteoconduction, potential osteoinductivity, and a capacity of being totally absorbed, it has been widely used in clinic as a local antibiotic carrier for treatment of chronic osteomyelitis in our hospital. So we hypothesized that one-stage surgery may be achieved to reconstruct small bone defects with CS as a spacer.
